# *Swertiamarin* Rescues 3-NPA-Induced Defective Follicular Development via Modulating the NRF2/HO-1 Signaling Pathway in Granulosa Cells

**DOI:** 10.3390/antiox14070794

**Published:** 2025-06-27

**Authors:** Luoyu Mo, Gan Yang, Dongju Liu, Huai Zhang, Xiaodong Dong, Fuyong Li, Ziqian Huang, Dini Zhang, Yan Xiong, Xianrong Xiong, Honghong He, Jian Li, Shi Yin

**Affiliations:** 1 College of Animal & Veterinary Sciences, Southwest Minzu University, Chengdu 610041, China; moluoyu20@163.com (L.M.); 18288246608@163.com (G.Y.); qingjiu001020@163.com (D.L.); zhanghuai0224@163.com (H.Z.); cehngzi040523@163.com (X.D.); boring74086952025@163.com (F.L.); 13730346520@139.com (Z.H.); azdn0285@163.com (D.Z.); xiongyan0910@126.com (Y.X.); xianrongxiong@163.com (X.X.); honghong3h@126.com (H.H.); 2 Key Laboratory of Qinghai-Tibetan Plateau Animal Genetic Resource Reservation and Utilization, Ministry of Education, Chengdu 610041, China; 3 Key Laboratory of Animal Science of National Ethnic Affairs Commission of China, Southwest Minzu University, Chengdu 610041, China

**Keywords:** *Swertiamarin*, oxidative stress, follicle development, granulosa cell, NRF2/HO-1 pathway

## Abstract

The normal development of ovarian follicles, characterized by oocyte growth and granulosa cell proliferation, is essential for maintaining female fertility. Elevated oxidative stress, resulting from various in vivo and in vitro factors, significantly impairs follicular development, ovulation, and overall female fertility. *Swertiamarin*, a naturally occurring iridoid terpenoid compound, exhibits multiple beneficial properties, including anti-hyperlipidemic, anti-diabetic, and antioxidant effects. This study investigates the impact of *Swertiamarin* on follicular development impairment induced by oxidative stress, using the commonly applied oxidant 3-nitrophthalic acid (3-NPA) in a murine model. Our findings indicate that *Swertiamarin* administration mitigates the adverse effects of 3-NPA on follicular development and ovulation. Further analyses reveal that *Swertiamarin* treatment partially enhances granulosa cell proliferation and inhibits apoptosis under oxidative stress in vivo and in vitro. Moreover, *Swertiamarin* reduces oxidative stress in ovaries and granulosa cells exposed to 3-NPA. The expression levels of key members of the NRF2/HO-1 signaling pathway, including nuclear factor erythroid 2-related factor 2 (*Nrf2*), heme oxygenase 1 (*Ho-1*), and superoxide dismutase 1 (*Sod1*), were upregulated following *Swertiamarin* supplementation in 3-NPA-treated ovaries and granulosa cells. In conclusion, the present study demonstrates that *Swertiamarin* can partially restore defective follicular development induced by oxidative stress via modulating the NRF2/HO-1 pathway in granulosa cells. These findings provide novel insights into the potential application of *Swertiamarin* in enhancing female reproductive health and offer a promising strategy for addressing reproductive damage caused by oxidative stress.

## 1. Introduction

The follicle is the fundamental structural unit in female mammals for maintaining fertility. It is a functional complex composed of oocytes and granulosa cells (GCs). A series of key molecular signals regulates the activation of primordial follicles into primary follicles. Follicular development primarily involves the rapid growth of oocytes and the proliferation of GCs, progressing from a single layer (primary follicles) to two layers (secondary follicles) and then to multiple layers (preantral follicles). Subsequently, under the influence of hormones such as follicle-stimulating and luteinizing hormones, the growing follicle develops fluid-filled cavities (antral follicle), while some GCs differentiate into cumulus cells, facilitating oocyte maturation [1,2,3]. The development of mammalian follicles is a complex, multicellular, and highly coordinated biological process. At each stage, a certain number of follicles undergo elimination and atresia, with only a limited number advancing to the next stage [4,5]. The selection of antral follicles is regulated by gonadotropins, whereas the development of growing follicles is influenced not only by genetic factors but also by the microenvironment maintained by GCs [6,7,8].

Reactive oxygen species (ROS) primarily include superoxides, peroxides, and free radicals derived from oxygen. These molecules are characterized by their small size and high chemical reactivity. ROS can be generated through various processes, including normal metabolism, exposure to toxic compounds, interactions with metal ions, and radiation [9,10]. Under normal physiological conditions, low levels of ROS play essential roles in cellular signal transduction, hormone secretion, and cell growth. However, excessive ROS levels can cause damage to lipids, proteins, and nucleic acids, leading to intracellular protein denaturation, enzyme inactivation, lipid peroxidation, and DNA degradation, ultimately resulting in cell apoptosis [11,12,13]. Research has shown that elevated ROS concentrations have a detrimental effect on follicular development. For instance, intraperitoneal administration of 12.5 mg/kg of the potent oxidizing agent 3-nitropropionic acid (3-NPA) in female mice significantly reduces ovarian weight, the number of growing follicles, and mature oocytes while markedly increasing the number of atretic follicles [14]. The emerging evidence suggests that natural antioxidants hold promise for mitigating reproductive oxidative damage, yet few studies have explored the therapeutic potential of iridoid terpenoids in this context.

Natural antioxidants are abundant in various species of Chinese herbal medicine. *Swertiamarin*, a secoiridoid glycoside, is extracted from the *Enicostemma genus*, specifically from *Enicostemma littorale* and *Enicostemma axillare*, both of which belong to the *Gentianaceae* family [15,16]. It was reported that the extraction rates of *Swertiamarin* from *Enicostemma axillare* and *Enicostemma littorale* are 0.4% and 2.12%, respectively [17,18]. This compound has been documented to have therapeutic effects on various diseases, including diabetes, hypertension, and neuropathy [19,20]. For instance, *Swertiamarin* has been shown to alleviate diabetic peripheral neuropathy in rat models by inhibiting the NOX/ROS/NLRP3 signaling pathway [21]. In addition, it has demonstrated efficacy in reducing oleic acid-induced lipid accumulation and oxidative stress in hepatic steatosis [22]. In rat models exposed to cigarette smoke, *Swertiamarin* has been found to mitigate collagen deposition in the prostate, alleviate oxidative stress, and reduce local inflammation [23]. Furthermore, it has exhibited protective effects against carbon tetrachloride-induced liver injury and inflammation, a benefit attributed to its antioxidant properties mediated through the NRF2/HO-1 signaling pathway [24]. Notably, in insulin-resistant GCs from patients with polycystic ovarian syndrome, *Swertiamarin* has been observed to enhance the secretion of estradiol and progesterone while upregulating the expression of genes associated with steroidogenesis, suggesting its potential role in follicular development [25]. To test this hypothesis, we established a mouse model of 3-NPA-induced oxidative stress, which disrupts follicular development by inducing excessive ROS production. Our study aimed to investigate whether *Swertiamarin* supplementation could alleviate 3-NPA-induced follicular abnormalities and elucidate the underlying molecular mechanisms involving NRF2/HO-1 pathway activation. This research provides novel insights into the potential application of *Swertiamarin* in promoting female reproductive health.

## 2. Materials and Methods

### 2.1. Animal Treatment and Sample Collection

All ICR mice utilized in this study were procured from Chengdu Dossy Experimental Animal Co., Ltd., Chengdu, China. A total of 124 three-week-old female ICR mice, with body weights ranging from 10 g to 12 g, were maintained at a controlled temperature of 22–26 °C under a 12 h light/dark cycle, with adequate food and water sources. Mice were excluded from the study if they exhibited pre-existing health conditions or technical failures during procedures. To examine the effects of *Swertiamarin* on follicle development, all experimental mice were randomly assigned to different groups and received intraperitoneal injections of normal saline solution (Shengao, Xianyang, Shaanxi, China), 3-NPA (Macklin, N814665, Shanghai, China), and different concentrations of *Swertiamarin* (YuanYe, S28017, Shanghai, China) based on their body weight. The groups were designated as follows: (1) the Control group, treated with normal saline solution (*n* = 23); (2) the NPA group, which received 12.5 mg/kg of 3-NPA (*n* = 23); (3) the NPA + LSE group, administered 12.5 mg/kg of 3-NPA in conjunction with 25 mg/kg of *Swertiamarin* (*n* = 23); (4) the NPA + MSE group, which received 12.5 mg/kg of 3-NPA, along with 50 mg/kg of *Swertiamarin* (*n* = 23); (5) the NPA + HSE group, treated with 12.5 mg/kg of 3-NPA and 100 mg/kg of *Swertiamarin* (*n* = 23); (6) the LSE group, administered 25 mg/kg of *Swertiamarin* (*n* = 3); (7) the MSE group, which received 50 mg/kg of *Swertiamarin* (*n* = 3); (8) the HSE group, treated with 100 mg/kg of *Swertiamarin* (*n* = 3). Both 3-NPA and *Swertiamarin* were pre-dissolved in a normal saline solution. In the Control group, injections were conducted once daily at 10:00 a.m. for a duration of 28 days. In the remaining four groups, 3-NPA was administered via intraperitoneal injection to the mice once daily for seven consecutive days. From day 8 to day 28, 3-NPA and varying concentrations of *Swertiamarin* were administered once daily at 10:00 a.m. and 2:00 p.m., respectively. To minimize confounders, cage positions were rotated weekly, and animal handlers were blinded to group identities during behavioral testing. The weights of the mice were recorded after the last injection. Peripheral blood was collected for the detection of hormone levels, and the mice were then euthanized. Both ovaries were isolated, weighed, and treated differently according to the requirements of subsequent experiments. Isoflurane anesthesia and adjunctive measures were implemented to minimize animal suffering. All experiments were conducted under the permit guidelines established by Southwest Minzu University, and all animal procedures were conducted according to the guiding principles of the Animal Care and Ethics Committee of Southwest Minzu University Animal Care and Use (Ethical protocol code: SMU-202501019, Approval Date: 13 January 2025). Experimental humane criteria followed the “Guideline of Assessment for Humane Endpoints in Animal Experiment” (ICS 03.120.20).

### 2.2. Cell Culture and Treatment

Primary mouse ovarian granulosa cells were obtained commercially from Beijing Biobw Biological Technology Co., Ltd. (Catalog No.: bio-132564, Beijing, China) and cultured in a commercial culture medium (Procell, CM-M050, Wuhan, China), containing DMEM/F12, 10% fetal bovine serum, 1% penicillin–streptomycin antibiotics, EGF, and insulin. GCs were passaged and cultured in cell culture plates. Upon reaching 60–80% confluence, the cells were treated with media containing different concentrations of 3-NPA (0, 5, 10, and 20 mM) or *Swertiarexin* (0, 10, 25, and 50 μg/mL). The cells were subsequently incubated at 37 °C with 5% CO_2_ for 48 h before additional experiments were conducted.

### 2.3. Histological Analysis

Ovaries were fixed in 4% PFA for 24 h before paraffin embedding, and 5 μm paraffin sections were attached to microscope slides for hematoxylin–eosin staining following the instructions of a hematoxylin–eosin kit (Solarbio, G1120, Beijing, China). The staining results were observed and photographed using a ZEISS microscope (Zeiss, LSM800, Baden-Württemberg, Oberkochen, Germany).

### 2.4. Superovulation and MII Oocytes Collection

Each mouse (*n* = 8 per group) was administered an intraperitoneal injection of 10 IU of Pregnant Mare Serum Gonadotropin (PMSG) (Solarbio, P9970, Beijing, China) on the 28th day to promote follicular development. Following a 48 h interval, each mouse received an injection of 10 IU of human Chorionic Gonadotropin (hCG) (Solarbio, YZ-1817, Beijing, China). The cumulus-oocyte complexes (COCs) were surgically extracted from the oviducts 12 h post-injection. The MII oocytes were collected after the removal of GCs, which was achieved by treating the COCs with 0.3% hyaluronidase (Solarbio, H8030, Beijing, China) for a duration of 2 to 5 min.

### 2.5. Detection of Estrogen and Progesterone Levels

Each mouse was administered an intraperitoneal injection of 10 IU of PMSG. Following a 48 h interval, each mouse received an injection of 10 IU of hCG. Blood samples were collected from the mice after the simultaneous estrus treatment, with a total volume of 1 mL obtained from each mouse. After standing at room temperature for 30 min, the sample was centrifuged at 5000 rpm for 10 min, and the supernatant was collected. Culture media containing GCs were collected after treatment with 3-NPA and *Swertiamarin* for 48 h and centrifuged at 3000 rpm for 20 min at 4 °C. The supernatant obtained from the experiment was collected for further analysis. The concentrations of estradiol and progesterone were quantified using two enzyme-linked immunosorbent assay (ELISA) kits (Meimian, MM-45704M1; MM-0546M1, Yancheng, Jiangsu, China) in accordance with the manufacturer’s protocols.

### 2.6. Immunohistochemistry

Ovarian sections were deparaffinized and incubated with primary antibodies at 4 °C overnight. The slides were then incubated with biotin-conjugated secondary antibodies at room temperature for 1 h. The sections were observed using a confocal microscope (Zeiss, LSM800, Baden-Württemberg, Oberkochen, Germany) after staining with 3,3′-diaminobenzidine (DAB) and hematoxylin (Solarbio, G1120, Beijing, China). The antibodies utilized are listed in Table S1.

### 2.7. Measurement of ROS Levels

The levels of ROS in mice ovaries were assayed using the Tissue Reactive Oxygen Species Detection Kit (Biorab, HR8835, Beijing, China). In brief, 1 mL of homogenization buffer was added for every 50 mg of tissue. The resulting mixtures were then centrifuged at 1000× *g* for 10 min at 4 °C. Subsequently, 190 μL of the supernatant was combined with 10 μL of the O13 probe. The mixture was incubated for 30 min at 37 °C in the dark, after which the fluorescence intensity was measured using a spectrophotometer (Thermo Scientific, Varioskan LUX 3020, Waltham, MA, USA) at an excitation wavelength of 510 nm and an emission wavelength of 610 nm. A total of 50 μL of the supernatant was extracted and diluted for protein quantification using a BCA Protein Assay Kit (Solarbio, PC0020, Beijing, China). The levels of ROS were expressed as fluorescence intensity per mg of protein.

For GCs, the levels of ROS were evaluated utilizing a Reactive Oxygen Species (ROS) Assay Kit (Beyotime, s0033s, Shanghai, China). In brief, cells were cultured in 24-well plates and incubated in a medium containing a 10 μM DCFH-DA probe at 37 °C for 20 min. Cells that were treated with Rosup (Beyotime, s0033s, Shanghai, China, diluted 1:1000 with DMEM) for a duration of 20 min and subsequently loaded with probes served as positive controls. Subsequently, the cells were stained with Hoechst (Beyotime, C1011, Shanghai, China), and fluorescence was observed using a fluorescence microscope (Zeiss, LSM800, Baden-Württemberg, Oberkochen, Germany).

### 2.8. Terminal Deoxynucleotidyl Transferase Mediated dUTP Nick End Labeling (TUNEL) Assay

A TUNEL assay was conducted in accordance with the protocol of the One Step TUNEL Apoptosis Assay Kit (KeyGEN, KGA1406, Nanjing, Jiangsu, China). Briefly, ovarian sections were deparaffinized, and Proteinase K was applied dropwise to each slide, followed by incubation at 37 °C for 30 min. For GCs, the cell slides were immersed in 4% PFA and fixed for 30 min at room temperature. Subsequently, PBS containing 1% Triton X-100 (Solarbio, T8200, Beijing, China) was added to the slides, allowing for permeation for 5 min at room temperature. A total of 50 µL of the TdTase solution was administered to each slide, after which the sections were placed in a humidified chamber for 60 min at 37 °C in the dark. Following this, 50 µL of Streptavidin–Fluorescein solution was added to each slide, and the slides were again placed in a humidified chamber for 30 min at 37 °C in the dark. The nuclei were subsequently re-stained with DAPI (Biosharp, BL105A, Beijing, China), and the slides were washed with PBS before being sealed. Finally, the slides were photographed under a fluorescence microscope (Zeiss, LSM800, Baden-Württemberg, Oberkochen, Germany).

### 2.9. RNA Extraction, cDNA Synthesis, and Real-Time PCR

Total RNA was extracted from the ovaries and GCs in each group using a TRIzol Reagent (Vazyme, R401, Nanjing, Jiangsu, China). RNA purity was assessed with an ultraviolet-visible photometer (Shimadzu, Bio-Spec-nano, Kyoto, Japan). Briefly, 1 mL of the TRIzol Reagent and steel beads were added to the ovaries of each group, thoroughly ground in a high-throughput tissue grinder, and centrifuged at 12,000 rpm for 15 min at 4 °C. The aqueous phase was transferred to a fresh centrifuge tube, followed by the addition of an equal volume of ice-cold isopropanol. Following 10 min incubation at room temperature, the samples were centrifuged at 12,000× *g* for 15 min at 4 °C. The resultant RNA pellet was washed twice with 75% ethanol and subsequently dissolved in 30 μL of nuclease-free water. The RNA purity and concentration were quantified using a UV-Vis spectrophotometer (BioSpec-nano, Shimadzu, Kyoto, Japan). Subsequently, cDNA was synthesized with the HiScript II Q RT SuperMix for qPCR (Vazyme, R223, Nanjing, Jiangsu, China) following the manufacturer’s protocol. Real-time PCR was conducted using a ChamQ SYBR qPCR Master Mix (Vazyme, Q311-01, Nanjing, Jiangsu, China), as previously described [26]. *Actb* was set as the reference gene, and the fold change in gene expression was quantified using the comparative C_T_ method. The primers utilized are detailed in Table S2.

### 2.10. Western Blotting

Proteins from the ovaries and GCs were extracted using the RIPA Lysis Buffer (Servicebio, G2002, Wuhan, China), which contained 1% phenylmethylsulphonyl fluoride (Servicebio, G2008, Wuhan, China), in accordance with the manufacturer’s instructions. Briefly, the mouse ovaries of each group were thoroughly ground into powder in mortars containing liquid nitrogen, and then, the powder was transferred to a centrifuge tube containing a RIPA lysis solution and lysed on ice for 40 min. Then, the centrifuge tubes were centrifuged at 12,000 rpm for 15 min at 4 °C, and the supernatant was taken to detect the concentration of protein. The protein concentration was quantified using the BCA Protein Quantification Kit (Oriscience, PD101, Chengdu, Sichuan, China), and a total of 20 μg of protein was loaded into each well. The samples were subjected to separation via 15% sodium dodecyl sulfate–polyacrylamide gel electrophoresis (SDS-PAGE) and subsequently transferred to polyvinylidene fluoride membranes (Millipore, Burlington, MA, USA). The membranes were blocked with 5% nonfat milk for 2 h and then incubated with primary and secondary antibodies. A BeyoECL Star Kit (Beyotime, P0018S, Shanghai, China) was used to visualize the immunoreactive bands. Images were captured with an Invitrogen iBright CL750 Imaging System (Thermo, Waltham, MA, USA). ACTB served as a protein-loading control. The antibodies utilized are listed in Table S1.

### 2.11. Cell Counting Kit-8 (CCK-8) Assay

GCs were seeded into 96-well plates at a density of approximately 1 × 10^4^ cells per well. Subsequently, the cell proliferation rate was assessed using a Cell Counting Kit-8 (CCK-8) Kit (MCE, HY-K0301, Shanghai, China) following the manufacturer’s instruction. CCK-8 utilizes a water-soluble tetrazolium salt, WST-8 ((2-(2-methoxy-4-nitrophenyl)-3-(4-nitrophenyl)-5-(2,4-disulfophenyl)-2H-tetrazolium)) to evaluate cell proliferation. The optical density (OD) value at 450 nm was determined using a spectrophotometer (Thermo Scientific™, 1530-00183, Waltham, MA, USA). The cell viability was calculated according to the manufacturer’s instructions.

### 2.12. Determination of MDA Content

Ovarian tissues were homogenized in an ice water bath and subsequently centrifuged at 8000 g for 10 min at 4 °C to collect the supernatant. GCs were seeded in six-well plates at a density of 5 × 10^5^ cells/mL. Following treatment with 3-NPA and different concentrations of *Swertiamarin*, the cells were collected and centrifuged at 1000 rpm for 10 min. The supernatant was then removed, and a small volume of PBS was added to the cell pellet. The cells were then disrupted using an ultrasonic cell crusher (JY92-IIN, SCIENTZ, Ningbo, Zhejiang, China) in an ice water bath. The MDA content was quantified using an MDA Content Assay Kit (Solarbio, BC0025, Beijing, China) following the manufacturer’s protocol.

### 2.13. EdU Staining

EdU staining on GCs with different treatments was conducted utilizing the BeyoClick™ EdU Cell Proliferation Kit with Alexa Fluor 488 (Beyotime, C0071S, Shanghai, China) according to the manufacturer’s instructions. Briefly, GCs were incubated with an EdU (10 mM final concentration) solution at 37 °C for 2 h. Subsequently, the cells were fixed in 4% PFA and permeabilized with 0.3% Triton X-100 (Solarbio, T8200, Beijing, China) at room temperature for 15 min. After being washed three times in wash buffer (PBS containing 3% BSA), a click reaction solution was added, and the cells were incubated in the dark for 30 min. The nuclei were then stained with 10 μg/mL Hoechst (Solarbio, C0031, Beijing, China) and imaged using a ZEISS microscope (Zeiss, LSM800, Baden-Württemberg, Oberkochen, Germany).

### 2.14. Statistical Analysis

GraphPad Prism (version 9.0) was used for all statistical analyses. The results were analyzed using one-way ANOVA (with Tukey’s multiple comparison test as the post-hoc test). All data are expressed as mean ± standard error (Mean ± SEM). All experiments were independently replicated at least three times, yielding consistent results. Statistical significance was defined as *p* < 0.05.

## 3. Results

### 3.1. Swertiamarin Administration Enhanced the Impaired Follicular Development and Ovulation Induced by 3-NPA in Ovaries

**Figure 1:**
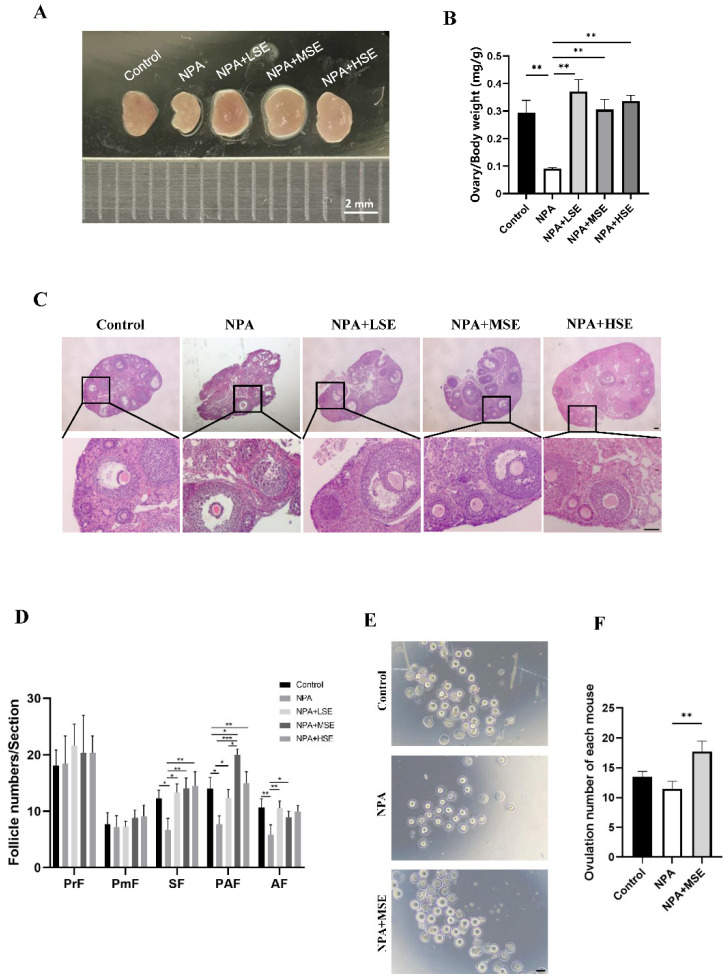
*Swertiamarin* treatment partially restored the impaired follicular development and ovulation in mice ovaries subjected to 3-NPA treatment. (**A**) Representative images of mouse ovaries of different groups. Control: mice injected with normal saline solution; NPA: mice injected with 12.5 mg/kg of 3-NPA; NPA + LSE: mice injected with 12.5 mg/kg of 3-NPA and 25 mg/kg of *Swertiamarin*; NPA + MSE: mice injected with 12.5 mg/kg of 3-NPA and 50 mg/kg of *Swertiamarin*; NPA + HSE: mice injected with 12.5 mg/kg of 3-NPA and 100 mg/kg of *Swertiamarin*. Scale bar: 2 mm. (**B**) The ratio of ovary weight to body weight (*n* = 3). (**C**) Histological sections of the ovaries were stained with hematoxylin and eosin. Scale bar: 500 μm (upper) and 50 μm (lower) (*n* = 3). (**D**) Quantitative analysis of follicles at different stages in ovarian sections. PmF: primordial follicle; PF: primary follicle; SF: secondary follicle; PAF: preantral follicle; AF: antral follicle (*n* = 3). (**E**) Representative images of MII oocytes from ovaries with different treatments (*n* = 8). Scale bar: 100 μm. (**F**) Mean number of MII oocytes collected from each female following superovulation with PMSG and HCG (*n* = 8). * *p* < 0.05, ** *p* < 0.01. and *** *p* < 0.001; one-way ANOVA (with Tukey’s multiple comparisons test as the post-hoc test).

The effects of different concentrations of *Swertiamarin* on normal follicle development were first assessed, and no significant changes in the follicle number were observed among the Control, LSE, MSE, and HSE groups (Figure S1). A model of ovarian dysfunction induced by 3-NPA was established to further evaluate the effects of *Swertiamarin* on defective follicular development. The administration of 3-NPA (the NPA group) resulted in a decreased ratio of ovarian to body weight (Figure 1A,B) and a significant reduction in the number of secondary, preantral, and antral follicles when compared to the Control group (Figure 1C,D). In contrast, the ratio of ovarian to body weight in mice treated with 25 mg/kg (the NPA + LSE group), 50 mg/kg (the NPA + MSE group), and 100 mg/kg (the NPA + HSE group) of *Swertiamarin* exhibited a significant increase relative to the NPA group (Figure 1A,B). Furthermore, although the numbers of primordial and primary follicles did not exhibit significant differences among different groups, *Swertiamarin* treatment resulted in a substantial increase in the number of secondary, preantral, and antral follicles, with an enhancement of at least 60.9% when compared to the NPA group (Figure 1C,D). Additionally, following priming with PMSG and HCG, the number of metaphase II (MII) stage oocytes recovered from the NPA + MSE group exhibited a 54.3% increase relative to the NPA group (Figure 1E,F). In conclusion, *Swertiamarin* effectively alleviated the impaired follicular development and ovulation induced by 3-NPA in mouse ovaries.

### 3.2. Swertiamarin Treatment Partially Improved Development and Function of GCs in 3-NPA-Treated Ovaries

**Figure 2:**
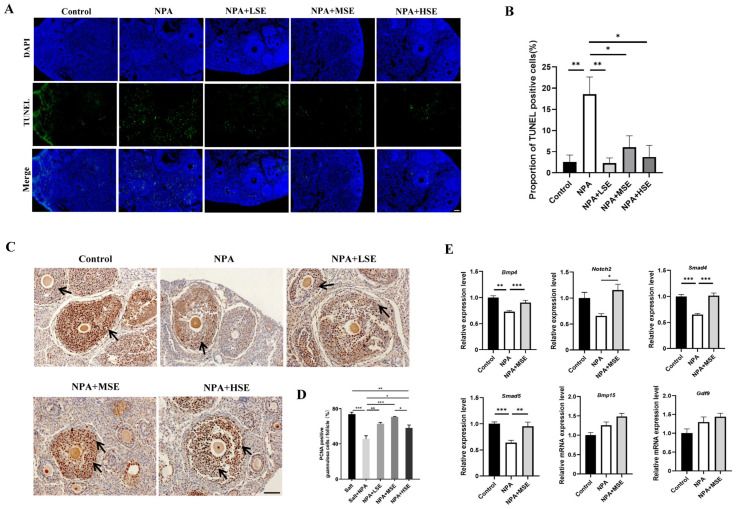
*Swertiamarin* promoted development and function of GCs in 3-NPA-treated mouse ovaries. (**A**) Detection of apoptosis by TUNEL assay in ovaries of different groups. Scale bar: 100 μm (*n* = 3). (**B**) Percentage of TUNEL-positive GCs in growing follicles in ovaries, as shown in (**A**). (**C**) Immunostaining for the cell proliferation marker PCNA in ovarian sections. PCNA-positive staining cells are marked by arrows (*n* = 3). Scale bars: 50 μm. (**D**) Percentage of PCNA-positive GCs in growing follicles, as shown in (**C**). (**E**) The relative mRNA expression levels of several oocyte and GC-specific genes associated with follicular development in ovaries subjected to different treatments (*n* = 3). * *p* < 0.05, ** *p* < 0.01. and *** *p* < 0.001; one-way ANOVA (with Tukey’s multiple comparisons test as the post-hoc test).

Given the essential roles of GCs in follicular growth and development, this study evaluated the proliferation and apoptosis of these cells in ovaries exposed to different concentrations of *Swertiamarin*. The TUNEL assay reveals that the proportion of apoptotic GCs in the ovaries of the NPA group was significantly greater than that observed in the Control group. In comparison to the NPA group, the proportions of apoptotic GCs in the NPA + LSE, NPA + MSE, and NPA + HSE groups were reduced by 87.8%, 67.2%, and 79.7%, respectively (Figure 2A,B). The proliferation of GCs was further validated through immunostaining for the cell proliferation marker proliferating cell nuclear antigen (PCNA) in the ovarian tissue sections. Compared to the NPA group, the proportion of PCNA-positive GCs in the NPA + LSE, NPA + MSE, and NPA + HSE groups exhibited increases of 38.2%, 55.0%, and 27.2%, respectively (Figure 2C,D). Furthermore, the mRNA expression levels of several critical genes involved in follicle development were evaluated. Although the expression levels of two oocyte-expressed genes, growth differentiation factor 9 (Gdf9) and bone morphogenetic protein 15 (Bmp15), did not exhibit significant differences between the NPA group and the NPA + MSE group. The expression levels of four GC-expressed genes—notch receptor 2 (Notch2), bone morphogenetic protein 4 (Bmp4), SMAD family member 4 (Smad4), and SMAD family member 5 (Smad5)—increased by 69.0%, 24.0%, 45.7%, 49.4%, respectively, in the NPA + MSE group compared to the NPA group (Figure 2E). Furthermore, considering that the majority of estrogen and a portion of progesterone are synthesized by GCs, the concentrations of estradiol and progesterone in the peripheral blood of mice subjected to different treatments were assessed. No significant differences were observed among the Control, NPA, and NPA + MSE groups (Figure S2). These findings indicate that the administration of *Swertiamarin* partially mitigated the impaired development and function of ovarian GCs in mice treated with 3-NPA.

### 3.3. Swertiamarin Ameliorated the Impaired Viability and Function of GCs Induced by 3-NPA In Vitro

**Figure 3:**
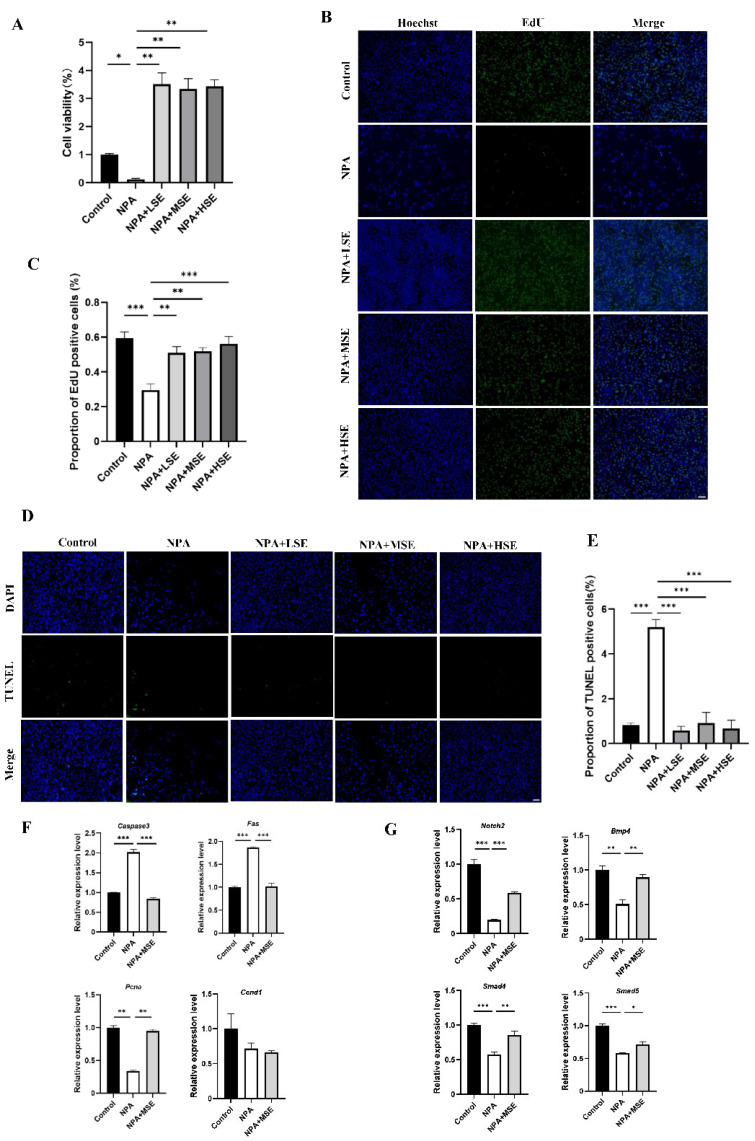
*Swertiamarin* treatment improved the viability of GCs treated with 3-NPA. (**A**) The CCK-8 assay was performed to detect the proliferation rates of GCs with different treatments (*n* = 3). (**B**) Proliferation detection by EdU staining in GCs with different treatments (*n* = 3). Bar: 100 μm. (**C**) Percentage of EdU-positive GCs in (**B**). (**D**) Apoptosis detection by TUNEL assay in GCs with different treatments (*n* = 3). Bar: 100 μm. (**E**) Percentage of TUNEL-positive GCs in (**D**). (**F**) Relative mRNA levels of several proliferation and apoptosis-related genes in GCs with different treatments (*n* = 3). (**G**) The relative mRNA expression levels of several follicle development-related genes in GCs subjected to different treatments (*n* = 3). * *p* < 0.05, ** *p* < 0.01. and *** *p* < 0.001; one-way ANOVA (with Tukey’s multiple comparisons test as the post-hoc test).

The impact of *Swertiamarin* on the proliferation of GCs under the treatment of 3-NPA was further validated by an in vitro model. The effects of different concentrations of *Swertiamarin* on the viability of GCs were first assessed, and no significant changes in viability were observed among the Control, LSE, MSE, and HSE groups (Figure S3). The results of the Cell Counting Kit-8 (CCK-8) assay and ROS level detection indicate that a concentration of 10 mM of 3-NPA significantly reduced cell viability and elevated the level of oxidative stress when compared to the Control group (Figure S4). In comparison to the 10 mM 3-NPA treatment alone (NPA group), the viabilities of GCs treated with 10 μg/mL (NPA + LSE group), 25 μg/mL (NPA + MSE group), and 50 μg/mL (NPA + HSE group) of *Swertiamarin* in combination with 10 mM of 3-NPA was significantly increased by at least more than 27-fold (Figure 3A). The TUNEL assay and EdU staining provided further evidence that *Swertiamarin* administration effectively alleviated apoptosis and improved the proliferation of GCs induced by 3-NPA (Figure 3B–E). Several genes associated with cell proliferation and apoptosis, including *Pcna*, cyclin D1 (*Ccnd1*), *Caspase3*, and the Fas cell surface death receptor (*Fas*), were analyzed. The mRNA levels of the pro-apoptotic genes *Caspase3* and *Fas* were found to decrease by 58.4% and 45.5%, respectively, in the NPA + MSE group compared to the NPA group. Conversely, the abundance of the proliferation-related gene PCNA increased by 178.1% in the NPA group relative to the NPA + MSE group (Figure 3F). Moreover, although the levels of estradiol and progesterone in the culture medium of GCs remained unchanged following different treatments (Figure S5), the expression levels of four genes associated with follicle development—*Notch2*, *Bmp4*, *Smad4*, and *Smad5*—increased by 202.0%, 76.7%, 48.8%, and 22.4%, respectively, in the NPA + MSE group compared to the NPA group (Figure 3G). These findings demonstrate that *Swertiamarin* ameliorated the impaired development and function of GCs induced by 3-NPA in vitro.

### 3.4. Swertiamarin Alleviates Oxidative Stress in Ovaries and GCs Treated with 3-NPA by Enhancing the NRF2/HO-1 Signaling Pathway

**Figure 4:**
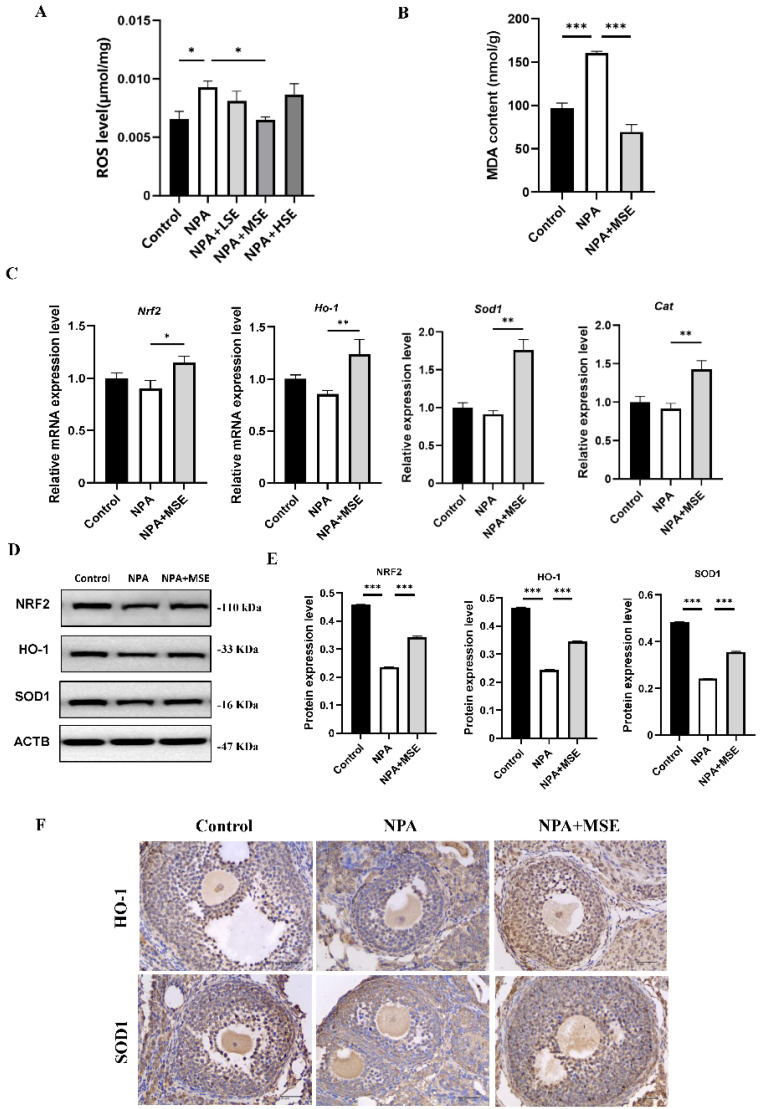
*Swertiamarin* attenuated 3-NPA-induced oxidative stress levels and enhanced NRF2/HO-1 pathway in mouse ovaries exposed to 3-NPA. (**A**) Detection of ROS levels in ovaries of different groups (*n* = 3). (**B**) Detection of MDA levels in ovaries (*n* = 3). (**C**) Relative mRNA levels of antioxidant genes *Nrf2*, *Ho-1*, *Sod1*, and *Cat* in ovaries of different groups (*n* = 3). (**D**) Protein expression of antioxidant proteins NRF2, HO-1, and SOD1 in ovaries of Control, NPA, and NPA + MSE groups (*n* = 3). (**E**) Optical density analysis of (**D**). (**F**) Immunostaining of HO-1 and SOD1 in ovarian sections (*n* = 3). Bar: 40 μm. * *p* < 0.05, ** *p* < 0.01. and *** *p* < 0.001; one-way ANOVA (with Tukey’s multiple comparisons test as the post-hoc test).

Considering that 3-NPA induces significant oxidative stress in mouse ovaries and that *Swertiamarin* is recognized as a potent antioxidant, we evaluated the levels of ROS in mouse ovaries following combined treatment with 3-NPA and *Swertiamarin*. Compared to the NPA group, ROS levels in the NPA + MSE group exhibited a decrease of 30.0% (Figure 4A). Additionally, malondialdehyde (MDA), a biomarker indicative of lipid peroxidation and oxidative stress, was significantly elevated in the NPA group relative to the Control group but exhibited a decrease in the NPA + MSE group (Figure 4B). The NRF2/HO-1 signaling pathway plays a crucial role in mediating cellular and tissue responses to oxidative stress. We assessed the mRNA expression levels of several components of the NRF2/HO-1 pathway, including nuclear factor erythroid 2-related factor 2 (*Nrf2*), heme oxygenase 1 (*Ho-1*), superoxide dismutase 1 (*Sod1*), and catalase (*Cat*), in the ovaries of the different experimental groups. In the NPA + MSE group, the expression levels of *Nrf2*, *Ho-1*, *Sod1*, and *Cat* increased by 27.7%, 44.8%, 92.2%, and 55.6%, respectively, when compared to the NPA group (Figure 4C). Correspondingly, the protein expression of NRF2, HO-1, and SOD1 exhibited a similar trend (Figure 4D,E). Additionally, the immunostaining results in the ovarian sections reveal that HO-1 and SOD1 displayed strong positive signals in GCs from both the Control and NPA + MSE groups, whereas expression was weak in the NPA group (Figure 4F).

**Figure 5:**
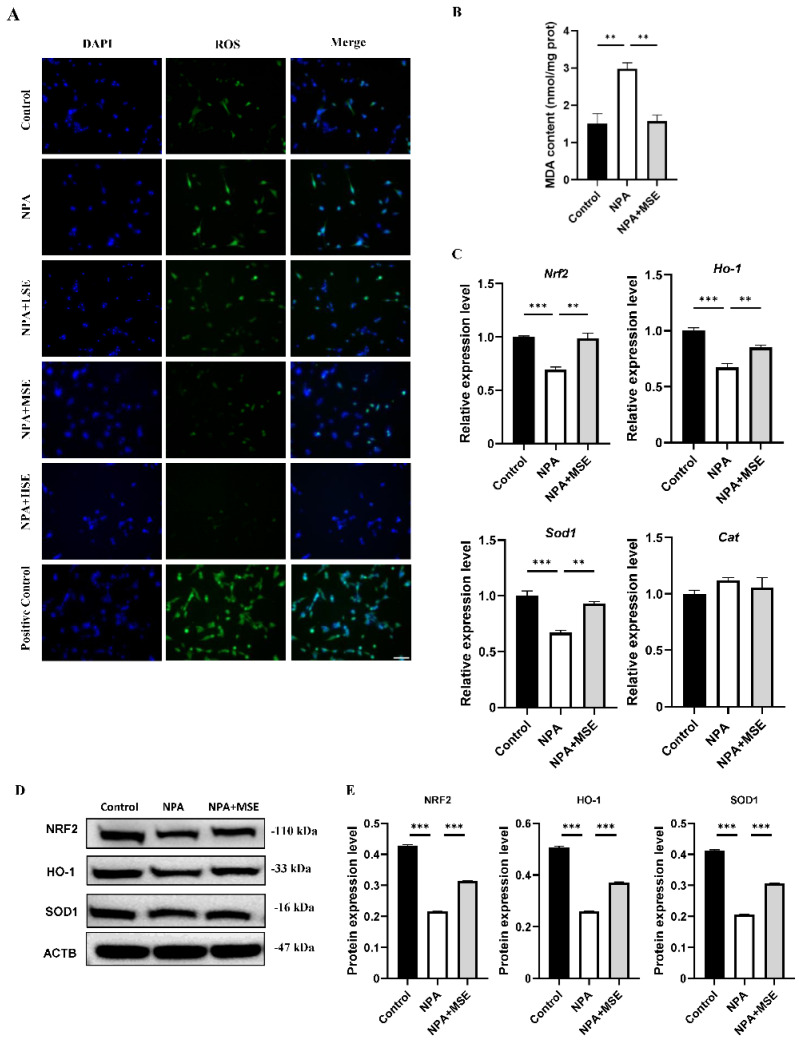
*Swertiamarin* treatment reduced oxidative stress and improved NRF2/HO-1 pathway in 3-NPA-treated mice GCs. (**A**) ROS level detection in mice GCs with different treatments. Bar: 50 μm (*n* = 3). (**B**) Detection of MDA levels in mice GCs (*n* = 3). (**C**) Relative mRNA levels of antioxidant genes *Nrf2*, *Ho-1*, *Sod1*, and *Cat* in GCs of Control, NPA, and NPA + MSE groups (*n* = 3). (**D**) Protein expressions of antioxidant proteins NRF2, HO-1, and SOD1 in ovaries of Control, NPA, and NPA + MSE groups (*n* = 3). (**E**) Optical density analysis of (**D**). ** *p* < 0.01. and *** *p* < 0.001; one-way ANOVA (with Tukey’s multiple comparisons test as the post-hoc test).

Consistent with the findings observed in ovarian tissues, the administration of *Swertiamarin* significantly reduced ROS and MDA levels in cultured GCs (Figure 5A,B). In comparison to the Control group, the mRNA expressions of *Nrf2*, *Ho-1*, *Sod1,* and *Cat* were significantly diminished in GCs treated with 3-NPA. Conversely, supplementation with *Swertiamarin* resulted in a significant increase in the expressions of *Nrf2*, *Ho-1*, and *Sod1* by 42.9%, 26.6%, and 39.1%, respectively, when compared to the NPA group (Figure 5C). Additionally, the protein expressions of NRF2, HO-1, and SOD1 in GCs subjected to different treatments were also assessed. The expression levels of these proteins were found to be suppressed following treatment with 3-NPA but were restored in the NPA + MSE group (Figure 5D,E). In conclusion, *Swertiamarin* alleviates oxidative stress in ovaries and GCs treated with 3-NPA by upregulating the NRF2/HO-1 signaling pathway.

## 4. Discussion

Excessive oxidative stress, both intracellularly and extracellularly, is a significant contributor to the abnormal development of ovarian follicles and the subsequent decline in female fertility capacity [27,28,29]. *Swertiamarin*, a natural iridoid terpenoid compound, demonstrates a variety of beneficial properties, including anti-hyperlipidemic, anti-diabetic, and antioxidant effects. This study investigates the protective effects of *Swertiamarin* on impaired follicular development induced by oxidative stress, thereby offering a novel strategy to solve reproductive damage induced by oxidative stress.

GCs are consistently involved in active proliferation and metabolic processes during follicular growth and exhibit heightened sensitivity to oxidative stress [30,31]. This sensitivity was evidenced by the suppression of secondary, preantral, and antral follicles following the administration of the oxidant 3-NPA in murine models. Subsequent supplementation with *Swertiamarin* resulted in a significant enhancement in the development of growing follicles, as well as a marked improvement in the survival capacity of GCs, both in vivo and in vitro. Additionally, the expression levels of several genes specifically expressed by GCs that regulate follicular development were restored, suggesting that *Swertiamarin* may alleviate defective follicle development under oxidative stress by enhancing the viability of GCs. However, given the critical role of oocytes in follicle development, it is interesting that *Swertiamarin* does not significantly enhance the expression of *Gdf9* and *Bmp15*, two oocyte-specific genes involved in follicular development in mice treated with 3-NPA. One possible explanation is that during follicular development, the oocyte, being in the meiotic arrest phase with lower metabolic activity and rich in antioxidants, exhibits lower sensitivity to changes in oxidative stress levels compared to the more metabolically active granulosa cells. The potential effect of *Swertiamarin* on the development and maturation of oocytes under conditions of oxidative stress warrants further investigation in subsequent studies.

Follicle development, progressing from secondary to preantral follicle stages, primarily depends on oocyte growth and GC proliferation. Estrogen and progesterone mainly regulate the maturation of antral follicles and ovulation [32,33,34]. Here, we found that the *Swertiamarin* and 3-NPA combined treatment significantly increased the number of antral follicles and mature oocytes in conditions of oxidative stress. However, *Swertiamarin* did not exhibit a significant impact on the levels of estrogen and progesterone secreted by GCs under oxidative stress, both in vivo and in vitro. This finding suggests that the promoting effect of *Swertiamarin* on the maturation of antral follicles and oocytes under oxidative stress is largely attributable to its facilitation of follicle development at earlier stages. Notably, *Swertiamarin* has been reported to increase the secretion of estrogen and progesterone in GCs of patients with insulin-resistant polycystic ovarian syndrome [25], indicating that *Swertiamarin* may exert inconsistent regulatory effects on steroid hormone synthesis in GCs under varying stress conditions.

The NRF2/HO-1 signaling pathway is acknowledged as a vital antioxidant mechanism that protects ovarian GCs from oxidative stress. The existing evidence indicates that the activation of NRF2 significantly increases the levels and activities of antioxidant enzymes, such as SOD and CAT, leading to a reduction in intracellular ROS production and GC apoptosis [35,36]. As a natural antioxidant, *Swertiamarin* has exhibited protective effects against carbon tetrachloride (CCl4)-induced liver injury and inflammation, attributed to its antioxidant properties via the NRF2/HO-1 pathway in rat models [24]. In the present study, we demonstrate that *Swertiamarin* effectively reduces oxidative stress and enhances the expression and activity of key components of the NRF2/HO-1 pathway, including NRF2, HO-1, SOD1, and CAT, in both ovaries and GCs. These findings provide evidence that *Swertiamarin* alleviates oxidative stress in GCs, at least in part, by upregulating the NRF2/HO-1 signaling pathway.

Numerous natural antioxidants have demonstrated efficacy in rescuing follicular development impairment in mice under oxidative stress. For instance, curcumin protects ovarian granulosa cells in vivo by regulating AMPK/mTOR-mediated autophagy [37], and proanthocyanidins counteract 3-NPA-induced premature ovarian failure (POF) through the activation of SESTRIN2-NRF2 oxidative stress responses [38], while icariin alleviates cisplatin-induced POF by ameliorating oxidative damage via activating the NRF2/ARE pathway [39]. Collectively, our work establishes *Swertiamarin* as a distinct antioxidant that rectifies 3-NPA-induced follicular impairment via activating the NRF2/HO-1 pathway in granulosa cells, a mechanism that diverges from the existing alternatives while offering novel therapeutic targets.

## 5. Conclusions

**Figure 6:**
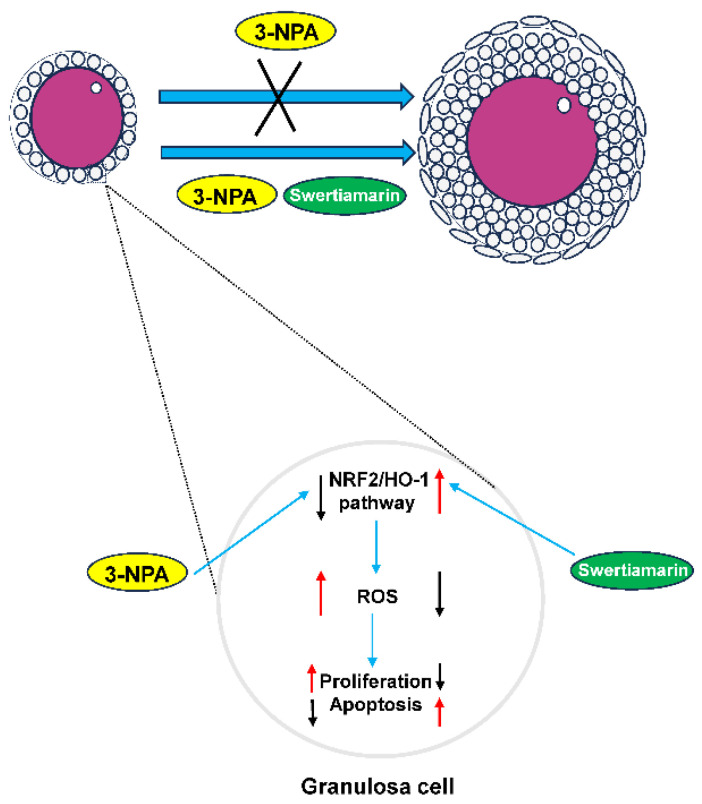
A schematic diagram of the proposed mechanisms by which *Swertiamarin* ameliorates 3-NPA-induced defective follicle development in mouse granulosa cells.

This study investigated the effects of *Swertiamarin* on impaired follicular development resulting from oxidative stress induced by 3-NPA. The results demonstrate that *Swertiamarin* can partially enhance follicle development by mitigating oxidative stress in GCs. The findings of this study indicate that *Swertiamarin* may influence female reproduction through the regulation of the NRF2/HO-1 signaling pathway (Figure 6). These results contribute valuable foundational data and suggest avenues for further investigation into the role of *Swertiamarin* in reproductive health.

## Data Availability

The data presented in this study are available upon request from the corresponding author. The data are not publicly available as these data are part of an ongoing longitudinal study. Full public release is deferred to preserve the integrity of future analyses.

## Supplementary Materials

The following supporting information can be downloaded at: https://www.mdpi.com/article/10.3390/antiox14070794/s1, Figure S1. The effect of different concentrations of *Swertiamarin* on follicle development in normal mice. (A) Histological sections of the ovaries were stained with hematoxylin and eosin. Control: mice injected with normal saline solution; LSE: mice injected with 25 mg/kg of *Swertiamarin*; MSE: mice injected with 50 mg/kg of Swertiamarin; HSE: mice injected with 100 mg/kg of *Swertiamarin*. Scale bar: 500 μm (upper) and 50 μm (lower) (*n* = 3). (B) Quantitative analysis of follicles at different stages in ovarian sections. PmF: primordial follicle; PFs: primary follicles; SFs: secondary follicles; PAFs: preantral follicles; AFs: antral follicles (*n* = 3). Figure S2. Progesterone (PROG) and estradiol (E2) levels in mice peripheral blood from Control, NPA, and NPA + MSE groups (*n* = 3). Figure S3. The effect of different concentrations of *Swertiamarin* on cell viability in normal GCs by CCK-8 assay (*n* = 3). Figure S4. Establishment of 3-NPA-induced oxidative stress model in mice GCs. (A) A CCK-8 assay was performed to detect the proliferation rates of mice GCs treated with different concentrations of 3-NPA (*n* = 3). (B) ROS level detection in mice GCs with different treatments. Bar: 100 μm. Figure S5. *Swertiamarin* treatment had no impact on progesterone and estradiol synthesis in culture medium of Control, NPA, and NPA + MSE groups of GCs (*n* = 3). Table S1. Antibodies. Table S2. Primer sequences.
