# Supplementary figures and images for: Swertiamarin Rescues 3-NPA-Induced Defective Follicular Development via Modulating the NRF2/HO-1 Signaling Pathway in Granulosa Cells
Source: Antioxidants (Basel). 2025 Jun 27;14(7):794. doi: 10.3390/antiox14070794 (PMC12291750; doi:10.3390/antiox14070794)

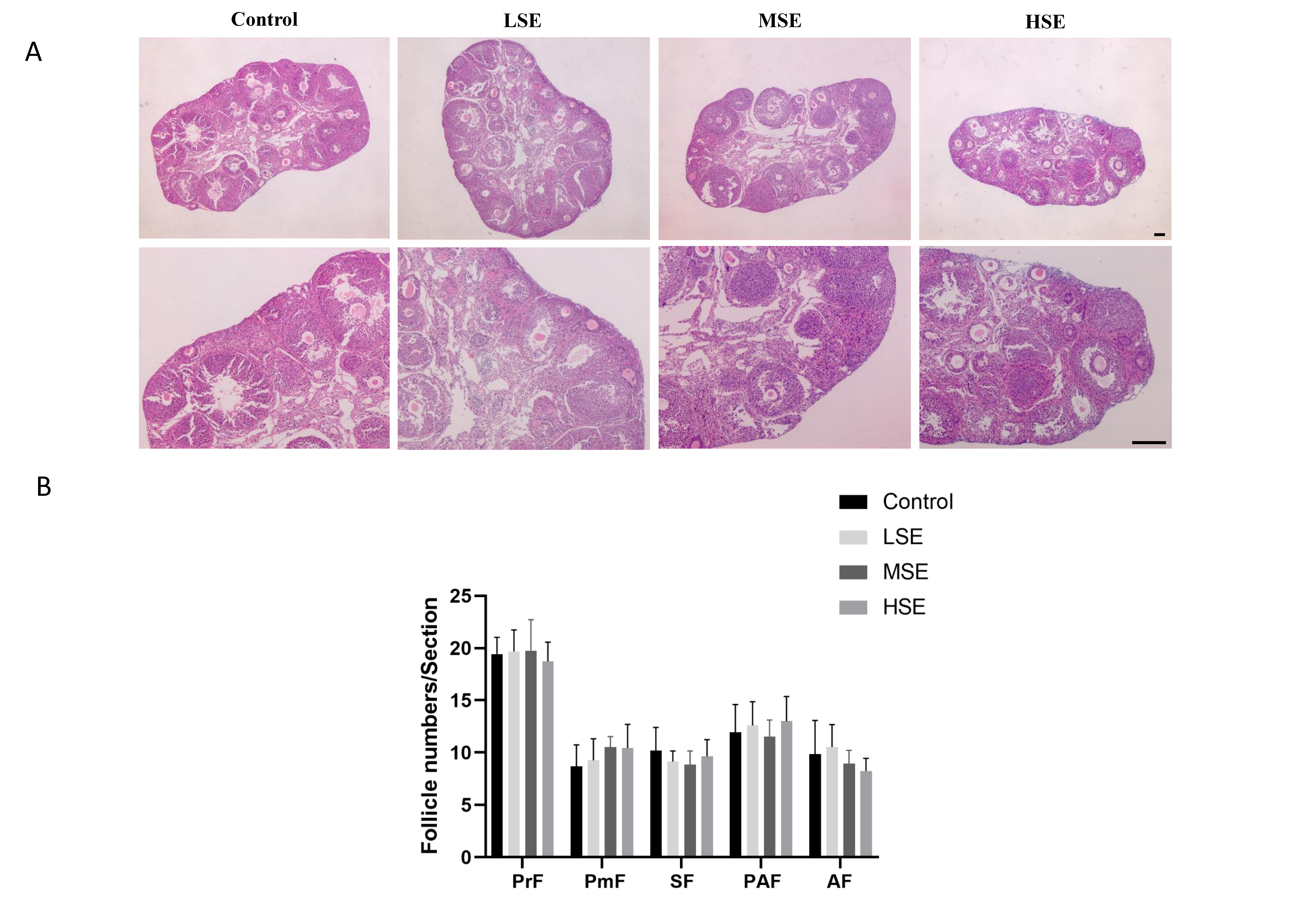

Supplement: Supplementary file 1 [file antioxidants-14-00794-s001.zip › Figure.S1.tiff]

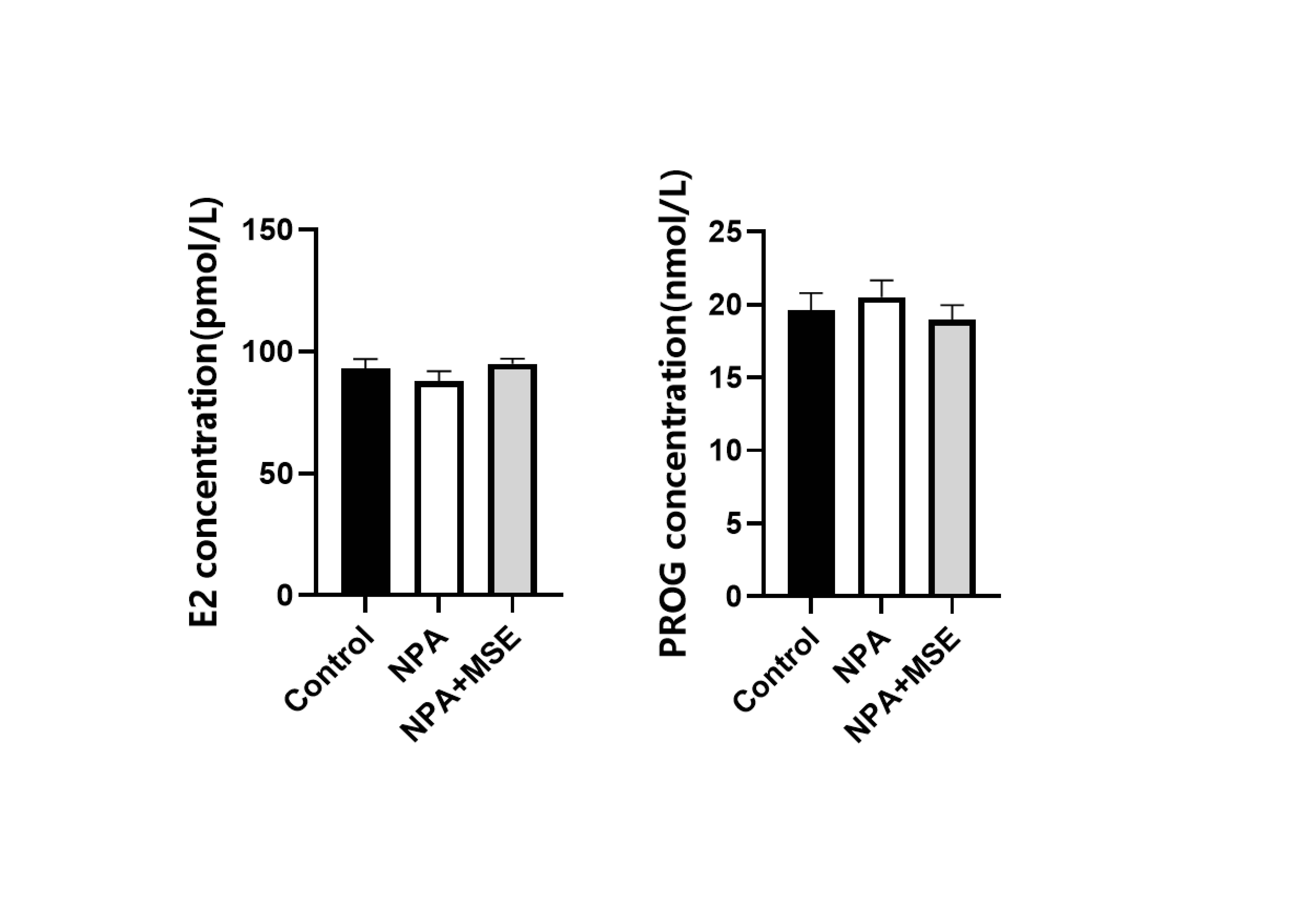

Supplement: Supplementary file 1 [file antioxidants-14-00794-s001.zip › Figure.S2.tiff]

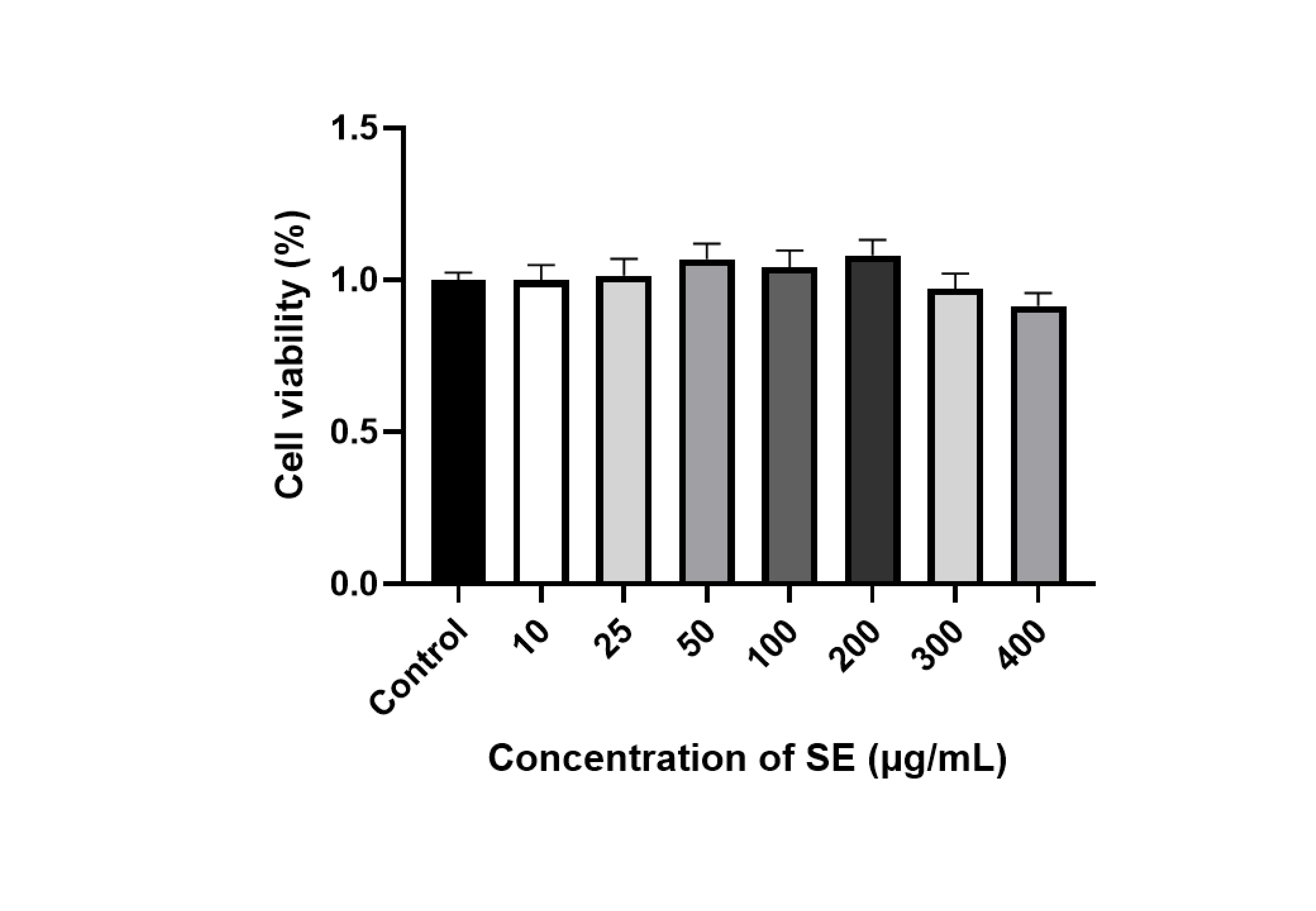

Supplement: Supplementary file 1 [file antioxidants-14-00794-s001.zip › Figure.S3.tiff]

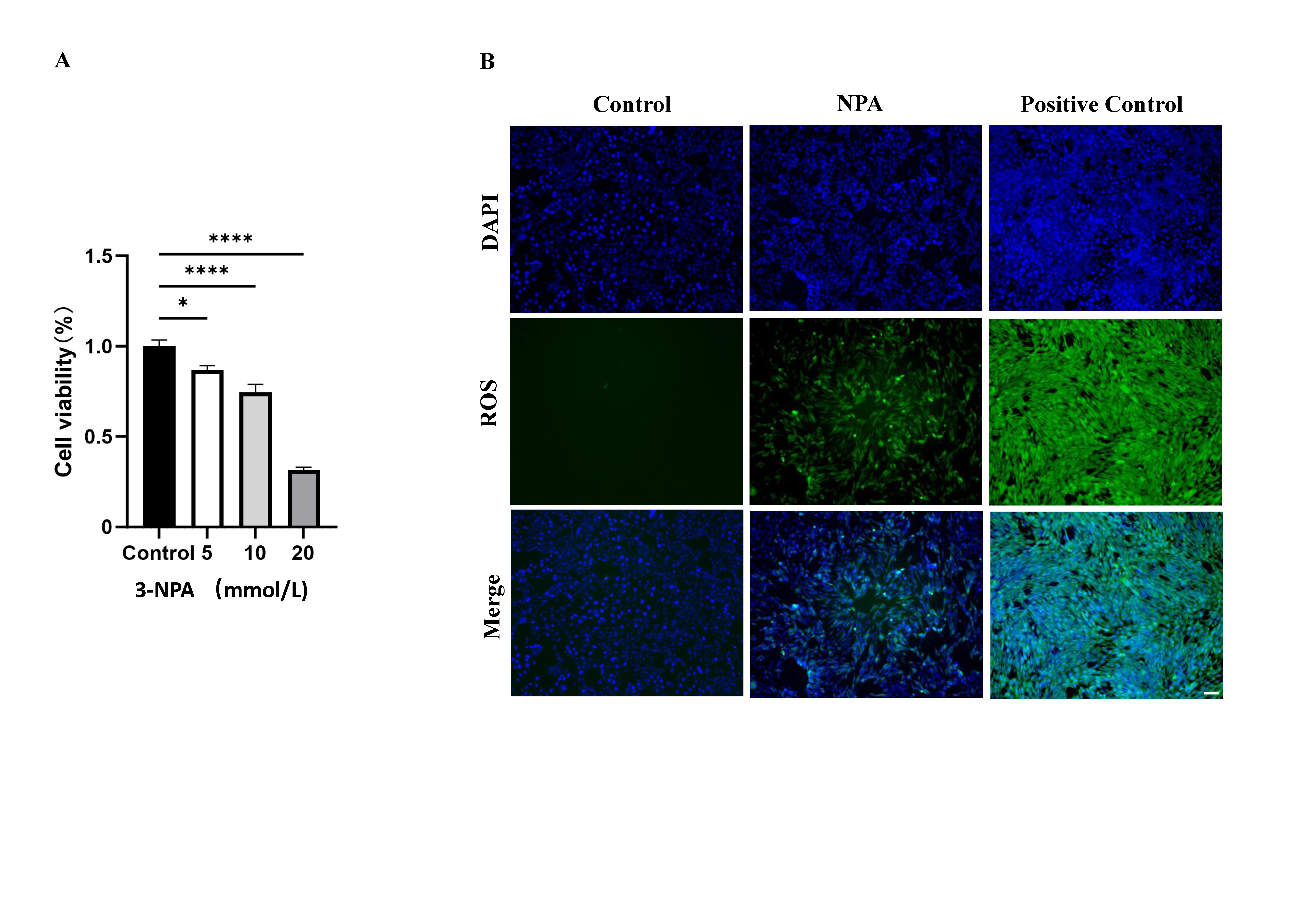

Supplement: Supplementary file 1 [file antioxidants-14-00794-s001.zip › Figure.S4.tiff]

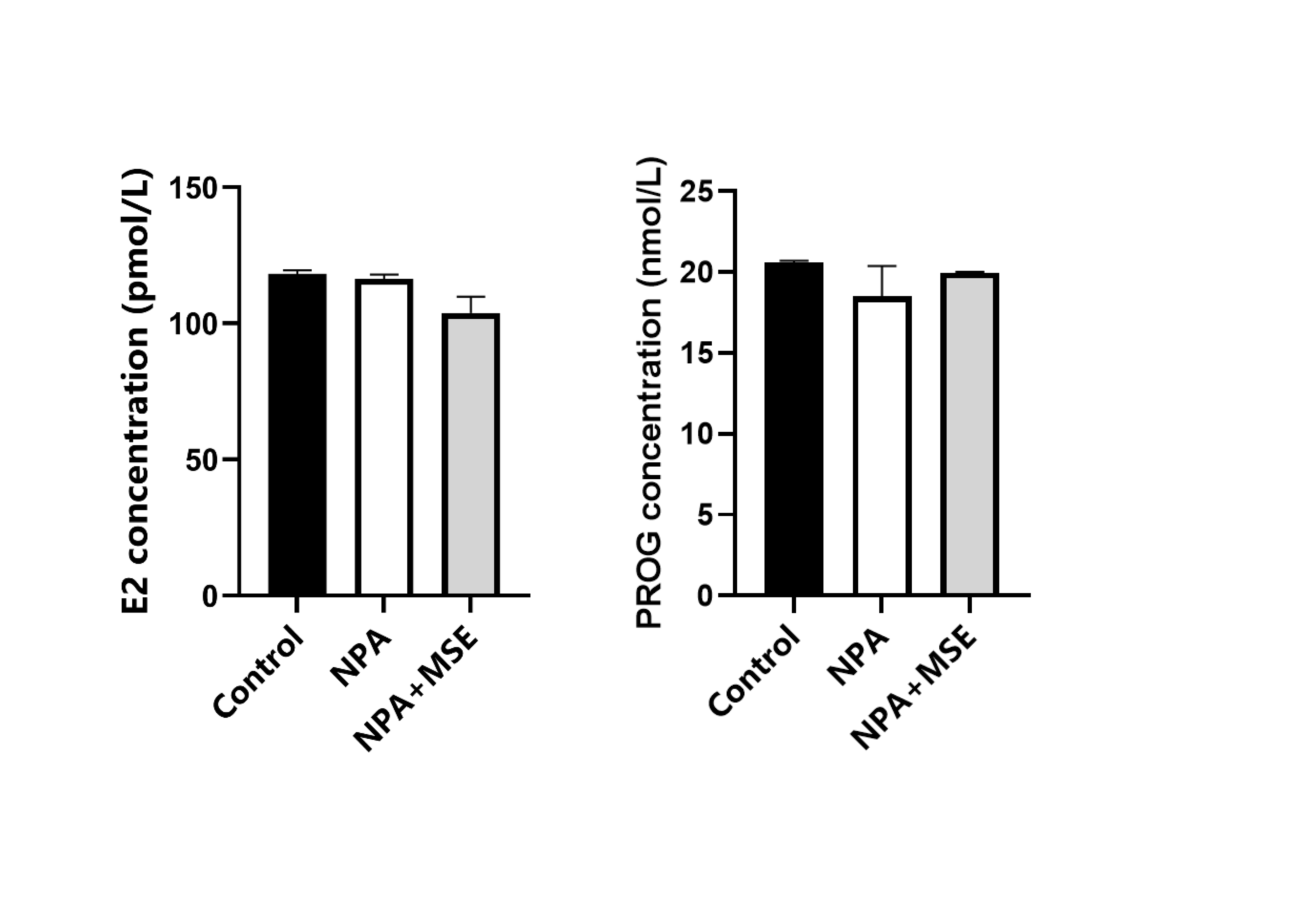

Supplement: Supplementary file 1 [file antioxidants-14-00794-s001.zip › Figure.S5.tiff]
